# Blood-Based Biomarkers of Aging and Neurodegeneration in Longitudinal Cohort Studies of Brain Aging

**DOI:** 10.1093/geroni/igaf122.208

**Published:** 2025-12-31

**Authors:** Natascha Merten, Josef Coresh, Bryan James

## Abstract

Alzheimer’s disease and related dementias (ADRD) have a long preclinical stage, with early pathological brain changes starting often in midlife, decades before any clinical symptoms. Identifying individuals at risk for cognitive and physical decline early will thus be most effective for intervention and prevention methods. Blood-based biomarkers are cost-effective and non-invasive and are promising for developing screening methods. To date, different blood-based biomarkers have been studied. Most of our knowledge stems from clinical study cohorts or studies enriched for patients with neurological diseases such as ADRD. Community- and population-based cohorts on the effectiveness of different biomarkers in the general population are rare. This symposium tries to fill this gap. We present work based on longitudinal data from the community-based Atherosclerosis Risk in Communities (ARIC) Study and the ACHIEVE trial as well as from the Beaver Dam Offspring Study-Neurocognitive Aging Study. We will show how different midlife and late life plasma-based biomarkers of neurodegeneration and AD are related to i) functional changes of cognition and ii) structural brain changes in cortical thickness later in life. Moreover, we will show how i) a combined risk prediction scores of plasma biomarkers of neurodegeneration and AD and ii) a blood-based biomarker of accelerated aging might help identify individuals at high risk for cognitive decline. This work will inform our understanding of the potential utility of these blood markers for a larger scale application in general population screenings for brain aging.

